# Evaluation of ANKOMMEN as a group intervention based on life story work for adolescents in residential care in Germany: a single-arm pilot study

**DOI:** 10.1186/s13034-024-00817-w

**Published:** 2024-10-22

**Authors:** Steffen Schepp, Jörg M. Fegert, Miriam Rassenhofer, Sara Regner, Andreas Witt, Elisa Pfeiffer

**Affiliations:** 1 Department for Child and Adolescent Psychiatry and Psychotherapy, University Hospital Ulm, Ulm, Germany; 2 German Center for Mental Health (DZPG), partner site Ulm, Ulm, Germany; 3 University Hospital of Child and Adolescent Psychiatry and Psychotherapy, University of Bern, Bern, Switzerland; 4 Department of Psychology, Catholic University Eichstätt-Ingolstadt, Eichstätt, Germany

**Keywords:** Residential care, Life story work, Adolescents in care, Group intervention, Child welfare

## Abstract

**Background:**

Adolescents face many challenges when coping with out-of-home placement, and life story work can be helpful in this context. Typically conducted in individual settings, life story work’s high resource requirements pose a challenge for implementation in the standard care of youth welfare institutions. To address this issue, the ANKOMMEN intervention was developed as a manualized group program for adolescents in residential care focusing on processing and coping with experiences associated with their out-of-home placement.

**Method:**

The intervention was evaluated in a single-arm pilot study with questionnaires administered at three time points (pre-intervention, post-intervention, and 3-month follow-up). The primary outcome was self-efficacy, while secondary outcomes included self-esteem, depressive symptoms, posttraumatic stress symptoms, and behavioral problems. A total of 31 intervention groups with 115 adolescents (*M* = 14.91 years; *SD* = 1.45; 52.2% male) were conducted between October 2020 and September 2022 in Germany. Data were analysed using mixed effect models.

**Results:**

Pre-post comparisons revealed increased self-efficacy (*d* = −0.80) and self-esteem (*d* = −0.68) among participants with below-average scores prior to the intervention. Additionally, there was a decrease in self-reported depressive symptoms (*d* = 0.76), self-reported posttraumatic stress symptoms (*d* = 0.58), self-reported internalizing behavior problems (*d* = 0.74), caregiver-reported internalizing behavior problems (*d* = 0.76), and self-reported externalizing behavior problems (*d* = 0.52) for participants with clinically relevant scores prior to the intervention. These improvements were stable in the 3-month follow-up assessment. Furthermore, the intervention proved its feasibility in standard care within the context of the evaluation study.

**Conclusions:**

The results of the pilot study provide preliminary evidence for the feasibility and potential effectiveness of ANKOMMEN but further research is needed to obtain valid evidence for the efficacy of the intervention.

**Supplementary Information:**

The online version contains supplementary material available at 10.1186/s13034-024-00817-w.

## Background

Numerous children and adolescents living in out-of-home care have a history of maltreatment and abuse, including physical, sexual, and emotional abuse, exposure to domestic violence, and neglect [1]. These distressing experiences often occur in the context of other adversities within the family of origin, such as poverty, parental mental or physical health issues, parental substance abuse, or parental delinquency [1–3]. Against this backdrop it comes as no surprise that approximately three-quarters of children and adolescents in residential care have experienced at least one potential traumatic life event in the past [4, 5]. In addition to psychosocial risk factors, biological risk factors (such as premature birth, prenatal exposure to noxious substances, and genetic predispositions to mental health issues), occur more frequently among children and adolescents in out-of-home care [2, 3]. As a result of the accumulation of these risk factors, children and adolescents in out-of-home care face a significantly higher likelihood of developing mental health problems than their peers in the general population [6–8].

While out-of-home care is intended to protect at-risk children and adolescents, it often involves additional stressors, including separation from significant attachment figures, loss of the familiar environment, feelings of guilt, conflicts of loyalty, and uncertainty about the future [9–11]. Given the multitude of stressors, pedagogical support alone is often insufficient to counteract the chronicity of behavioral and emotional problems [12–14]. Particularly during the initial year of placement, the mental health development of children and adolescents is crucial for their future prognosis, as the persistence of behavioral problems increases the likelihood of placement instability [14]. Since more frequent placement changes are associated with further exacerbations of behavioral problems, affected children and adolescents are at risk of finding themselves in a vicious circle [15–18]. A biography characterized by frequent placement changes is linked with poorer mental and physical health outcomes [19, 20], increased delinquency [21], and lower social participation [22] in later life. These findings underscore the importance of evidence-based interventions tailored to the needs of children and adolescents in out-of-home care. Early interventions targeting population-specific risk and protective factors could help prevent disruptions in care, and alleviate the mental health burden in this vulnerable population. Life story work is one approach to addressing the special needs of children and adolescents in out-of-home care.

### Life story work as a support method

Life story work is a method that aims to develop a coherent narrative of a person’s life through guided reflection on personal experiences [23]. Reflecting on their behavior through an autobiographical narrative allows individuals to link life events to personal characteristics. This facilitates the development of a cohesive sense of self amid a changing environment [23]. In this way, life story work helps bridge a person’s past, present, and future, and fosters the formation of a cohesive identity [24]. For children and adolescents in out-of-home care, whose biographies often present many discontinuities, establishing and maintaining a sense of continuity of self over time can prove to be particularly challenging. This is because frequent changes and experiences of loss tend to reinforce the impression that nothing is permanent, including their own identity, and that their impact on the environment is very limited [25]. Moreover, childhood adversity is linked to further disruptions in identity development (see [26]). Consequently, adolescents in care are at a heightened risk of dysfunctional identity development and the associated internalizing and externalizing behavioral problems [26–29]. Adolescents in out-of-home care in particular could, therefore, benefit from the implementation of life story work in child and youth welfare settings.

Unfortunately, only a few scientific studies have evaluated the effects of life story work on the mental health and well-being of children and adolescents in out-of-home care. In their systematic review Hammond, Young and Duddy [30] identified 24 predominantly qualitative studies on the effectiveness of life story work for children and adolescents in care. The included studies showed a positive impact of life story work on identity development [31–33], self-esteem [33], on the capacity to deal with emotional and behavioral challenges, and on the improvement of relationship quality [31, 34, 35]. However, the review also highlighted considerable heterogeneity in the quality of the implementation of life story work due to the lack of consistent quality standards and adequate training programs based thereon (see [30]). The lack of quality standards and the high demand for the human resources needed for life story work in individual settings constitute major barriers to their implementation in the standard care of youth welfare institutions.

Manualized group sessions offer a potential solution to these problems as they provide a framework for implementing life story work in a standardized and resource-efficient manner. However, there is a lack of scientifically evaluated standardized interventions and meaningful data regarding the effects of standardized life story work in group settings for adolescents in out-of-home care. The ANKOMMEN (German word for “arriving”) intervention was developed to address this research gap and to improve the care of adolescents living in youth welfare institutions.

### Concept of ANKOMMEN

Based on the rationale of life story work described above, the ANKOMMEN intervention was developed to help participants in processing the experiences related to their out-of-home placement and integrating them into their biographies by bridging their past, present, and future. The intervention also provides targeted support for dealing with challenges associated with out-of-home care, such as conflicts of loyalty, stigmatization, and uncertainty about the future. In this context it is important to clarify that ANKOMMEN is not a trauma-specific intervention as it focuses on coping with the consequences of living in residential care and accepting this situation, rather than on coping with traumatic life experiences in particular. However, the intervention may also have secondary effects on post-traumatic symptomatology due to its narrative approach on life events associated with out-of-home placement. By fostering the development of a coherent and self-strengthening narrative of the out-of-home placement, improving coping strategies for placement-associated issues, enhancing self-efficacy, and self-esteem, the intervention aims to reduce the global mental health burden among participating adolescents and, by extension, improve the overall prognosis of the out-of-home placement.

In particular, an improvement in self-efficacy seems to be crucial for the overall prognosis of out-of-home care. Self-efficacy describes the positive appraisal of a person’s ability to cope with challenging situations and overcome associated barriers to goal achievement [36, 37]. In several studies, self-efficacy was found to be one of the most important protective factors for children at risk, both generally and more particularly in the context of child abuse and neglect [38–41]. There is convincing evidence that perceived self-efficacy can at least partially buffer the negative effects of abuse and neglect, and that victims of such experiences with higher self-efficacy have fewer mental health issues and a higher quality of life [42–44]. With this in mind, addressing self-efficacy in interventions in standard care could be highly beneficial for children and adolescents in out-of-home placement. Life story work could contribute to the improvement of self-efficacy by supporting participants in exploring their history, reflecting on difficulties they have overcome so far, and integrating this self-strengthening narrative into their identity concept. The gain in awareness that they will probably be able to overcome future challenges too, could facilitate access to more comprehensive coping strategies, and this could hone their ability to deal with difficult situations and emotions [45].

Self-esteem can be defined as the global appraisal of a person’s value-based beliefs about themselves [46]. High self-esteem can be an effective protective factor, whereas low self-esteem is associated with higher rates of mental and physical illness, increased rates of delinquency, substance abuse, and lower academic success [46, 47]. In the context of the intervention, self-esteem can be enhanced in many ways. First, there is a major association between the enhancement of self-esteem and self-efficacy: A consciously perceived high level of self-efficacy in significant domains, often resulting from past achievements, can strengthen a person’s self-esteem. In turn, high self-esteem influences in a positive manner how a person spontaneously interprets a situation and how they appraise and choose coping strategies [46, 48]. This increases the likelihood of experiencing success again and creates a positive feedback loop. In this way, self-esteem and self-efficacy, and the explained relation between them play an important role in a main objective of life story work: to build a bridge between a person’s past, present, and future, and thus to develop a cohesive identity. Second, the combination of life story work and a group setting offers unique possibilities for enhancing participants’ self-esteem. The disclosure of personal and even stressful experiences associated with the out-of-home placement in the group can be conducive to creating a sense of connectedness among the participants because most of them have probably had similar experiences. Together with other well-known general positive effects of intervention groups, such as group cohesion and the normalization of adverse thoughts and feelings [49–52], the mutual social support during the stressful confrontation with a person’s history in the course of the intervention is particularly suited to producing a positive effect on the self-esteem of both the person receiving and the person giving support [53–55].

There are some programs designed to support the well-being and mental health of children and adolescents in out-of-home care through specific training of their caregivers, such as the Ripple Project [56] or the Connect for Kinship Parents Project [57]. However, only a few interventions are applied directly to children and adolescents in out-of-home care and specifically promote coping with the consequences of the out-of-home placement. For instance, the Fostering Healthy Futures program [58] uses a mentoring and skills group approach to support children in foster care by building resilience and coping skills. While the program has demonstrated its effectiveness [59], it poses challenges for implementation in standard care due to its 30-week duration and high resource requirements. Another example is the DREAMR project, which incorporates elements of life story work into its intervention framework but failed to demonstrate sufficient effectiveness [60]. In contrast, the ANKOMMEN intervention is specifically designed as a resource-efficient group intervention based on the rationale of life story work for adolescents in residential care. However, this unique approach, to the best of our knowledge, needs to prove its feasibility and effectiveness before further dissemination can be considered.

To this end, the intervention was evaluated in a pilot study using a mixed-methods design to assess all of its potential effects, both in standardized questionnaires and on an individual level. This article only refers to the quantitative analyses of the pilot study. The results of the qualitative analyses of interviews with participants have been published elsewhere [61]. The primary outcome of the study presented here was self-efficacy, as it is rooted in the rationale of life story work and is of outstanding importance for predicting outcomes in out-of-home placements. As secondary outcomes, we selected self-esteem and mental health difficulties, specifically posttraumatic stress symptoms (PTSS), depressive symptoms, and behavioral problems, that are highly prevalent in this population [6–8]. Additionally, we collected data about the feasibility and treatment fidelity of the intervention to evaluate their applicability in standard care.

Based on the rationale of the intervention and of life story work, we hypothesized that participation in the ANKOMMEN intervention increases self-efficacy and self-esteem in adolescents, decreases self-reported depressive symptoms, self-reported and caregiver-reported PTSS and behavioral problems, and that these improvements persist three months after the completion of the intervention. In this context, we also investigated whether there are differences in improvements among participating adolescents with high and low mental health burden prior to the intervention. This exploratory research question arose from a considerable uncertainty expressed by our cooperating youth welfare institutions during the development of ANKOMMEN as to whether the intervention is also suitable for adolescents with high mental health burdens and whether it might exacerbate their symptoms or compromise safety. We also hypothesized that the feasibility and treatment fidelity of the intervention in standard care settings are high, owing to the collaborative development and implementation of the intervention with staff in the collaborating youth welfare institutions.

## Methods

### Trial design

The single-arm pilot study was carried out in close collaboration with 17 youth welfare institutions located in southern Germany. For data collection, we used the online tool EQUALS [62]. The participants were invited to a pre-intervention screening with questionnaires before the first intervention session. The screenings took place in the youth welfare institutions, were conducted via tablets, and were supervised by staff of the study center. Due to COVID-19 pandemic restrictions, some of the screenings had to take place without any on-site supervision and were instead supervised via online meetings. After the intervention, participants were invited to a second screening (post-intervention follow-up). A third screening was conducted three months after the second screening (3-month follow-up). For each screening the adolescent participants received a €15 voucher. We collected caregiver reports of the same questionnaires, if available, from their primary caregivers parallel to the screening sessions of the adolescents. The study was approved by the ethics committee of the University of Ulm in February 2020 (reference number 417/19), and the data presented here were collected between October 2020 and September 2022.

### Recruitment

Youth welfare institutions located near the study center were recruited for collaboration through an official contact list of youth welfare institutions in southern Germany. Additionally, we recruited some of the collaborating institutions by utilizing a contact list provided after a scientific symposium where the idea of developing the intervention was discussed. We applied no inclusion criteria for the collaborating youth welfare institutions, resulting in a wide spectrum of sizes and pedagogical concepts among them. Each institution was required to designate a coordinator responsible for recruiting study participants, organizing the screenings within their institution, and maintaining communication with the study center. We trained all study coordinators before recruitment and provided ongoing support throughout the process. All participants of the intervention were recruited from our 17 cooperating institutions that participated in the development of the intervention. We only have information about the adolescents who were reported to us by the study coordinators. However, the total number of adolescents living in all cooperating institutions together was higher than the number of participants in the study. The study participants were recruited between September 2020 and May 2022.

### Participants

Prior to enrollment in the study, all participants and their legal guardians were given detailed information about the study procedures and the intervention, and they were required to provide informed written consent. To qualify for inclusion in the study, participants had to be (1) within an age range of 12 to 17 years as the intervention was designed for this age group and (2) their planned stay in the current youth welfare institution had to extend over at least three additional months to allow them to finish the intervention. Adolescents with acute suicidal ideations were excluded from the study because these adolescents needed a psychiatric first-line treatment for their acute suicidality before participating in our intervention. Additionally, the ANKOMMEN intervention was designed for implementation by social workers without clinical experience, aiming to enhance its feasibility in standard care. Therefore, participation in the intervention, which involves dealing with their own out-of-home placement and associated burdens, requires a certain degree of psychological stability from the participants. Acute suicidality was screened using item 9 (“Thoughts that you would be better off dead or of hurting yourself in some way”) of the Patient Health Questionnaire-9 [63], items 18 (“I intentionally hurt or tried to kill myself. “) and 98 (“I intentionally hurt or tried to kill myself. “) of the Youth Self Report [64], and item 9 (suicidal ideation) of the Beck Depression Inventory-II [65] as trigger for the subsequent more detailed assessment of acute suicidality using the Columbia-Suicide Severity Rating Scale [66]. Adolescents with acute suicidal ideations were advised to seek support from psychiatrists or psychotherapists, and further actions to ensure appropriate support were coordinated between the youth welfare institution and the clinical advisor at the study center.

### Development, content and implementation of ANKOMMEN

ANKOMMEN is a manualized group program based on the rationale of life story work for adolescents aged 12 to 17 years living in residential youth welfare institutions. It was developed at the Department for Child and Adolescent Psychiatry/Psychotherapy at University Hospital Ulm in cooperation with 17 residential youth welfare institutions in southern Germany. To ensure the intervention’s applicability in standard care settings, continuous feedback from the collaborating youth welfare institutions was sought during its development between October 2019 and July 2020. Additionally, focus groups with children and adolescents in residential care were conducted in the initial stages of development to gain valuable insights into their specific needs and preferences [67].

The resulting intervention is manualized and consists of eight 90-minute group sessions, with up to eight adolescent participants. These sessions are conducted weekly by two specifically trained staff members from the responsible youth welfare institution. As part of the evaluation study, the group leaders received comprehensive training in life story work and intervention implementation from the developers of the intervention, who are licensed child and adolescent psychotherapists and researchers in this field. Prior to training the staff members of the youth welfare institutions, the trainers conducted two intervention groups themselves to test and optimize the intervention materials. Furthermore, experienced psychotherapists from the Department for Child and Adolescent Psychiatry/Psychotherapy at University Hospital Ulm provided case consultations after each group session. The intervention follows a structured approach, divided into three consecutive phases. The initial two sessions focus on imparting knowledge about the reasons, aims, and procedures of out-of-home care in Germany, as well as providing guidance on emotion regulation strategies. Building confidence among the participants and preparing them to address personal and challenging issues in subsequent sessions are also key objectives during this phase. The following four sessions focus on facilitating biographical reflection regarding the out-of-home care experiences through guided writing of a narrative of the out-of-home placement, sharing it with the group, and addressing strategies to cope with associated issues such as conflicts of loyalty and stigmatization in daily live. The final two sessions are dedicated to reinforcing resources, teaching problem-solving strategies, and nurturing positive future perspectives. The specific content of each session is presented in Table 1. The participants are given individual workbooks to document their personal intervention progress. The intervention manual and workbook in German language can be made available upon request and will soon be published open access by a publishing house.

**Table 1:** Content of the ANKOMMEN intervention

Session number	Session name	Content
1	My rights	Introduction of group leaders, participants and intervention contents; joint development of group rules; learning more about children’s rights, reasons, procedures, and goals of out-of-home care
2	Emotions	Psychoeducation regarding the functions and differentiations of emotions; association between emotions, cognitions, and behavior
3	Introduction to life story work	Rational of life story work, personal fact sheet, map of places of residence up to present time
4	My story	Written story of participants’ out-of-home placement and first days in the current institution
5	Between two chairs	Learning how to deal with conflicts of loyalty
6	My official story	Pros and cons of dealing openly with the participants’ stories of their out-of-home placement; self-confident behavior; development of an official version of their own story of their out-of-home placement
7	Pen pals	Writing a letter to an imaginary person arriving in the participant’s youth welfare institution
8	My future	Learning problem-solving strategies; wishes and goals for the future; reflection of the intervention; closing ceremony

The intervention was delivered by 45 social workers (male *n* = 10, 22.2%) with a mean age of 35.33 years (*SD* = 10.91; ranging from 22 to 57 years). On average, they had 10.95 years of work experience (*SD* = 8.15, ranging from 0.83 to 30.00 years) and 8.06 years of experience in their current institution (*SD* = 6.95, ranging from 0.83 to 26.00 years). Out of the 45 social workers, *n* = 26 (51.0%) had graduated from university with at least a Bachelor’s degree, *n* = 19 (37.3%) had received professional educational training, and *n* = 23 (45.1%) had an additional qualification.

## Primary outcome

### General self-efficacy scale (GSE)

The German version of the General Self-Efficacy Scale (GSE; [36]) was used to measure the optimistic assessment of a person’s own possibilities for action in the face of challenging situations and barriers to action. Participants rate a total of 10 items on a 4-point Likert scale from 1 = “not at all true” to 4 = “exactly true”. Participants can achieve a maximum sum score of 40, with a sum score below 26 indicating self-efficacy below average in German adolescents [68]. Sum scores were utilized for the analyses. Internal consistency in our sample was good (Cronbach’s α = 0.87).

### Secondary outcomes

#### Achenbach scales (YSR / CBCL)

To assess behavioral problems, the German versions of the Youth Self Report (YSR; [64]) and the Child Behavior Checklist (CBCL; [69]) were used. With 119 items for the self-report (YSR) and 120 items for the caregiver report (CBCL), they measure internalizing and externalizing behavior problems and a total problem behavior score on a 3-point Likert scale ranging from 0 = “never” to 2 = “often”. T-scores equal to or above 60 indicate clinically relevant behavioral problems [70]. T-scores were utilized for the analyses. Internal consistency for the total problem behavior score in our sample was very good for the YSR (Cronbach’s α = 0.95) as well as for the CBCL (Cronbach’s α = 0.94).

### Child and adolescent trauma screen version 2 (CATS-2)

To assess PTSS, the German version of the Child and Adolescent Trauma Screen Version 2 (CATS-2; [71]) was used. The questionnaire consists of an event checklist of 15 potentially traumatic life events. Events with a “yes” response are then further investigated using 20 items on a 4-point Likert scale ranging from 0 = “never” to 3 = “almost always”, measuring PTSS criteria of the DSM-5 [72] and ICD-11 [73]. There was no further investigation of PTSS symptoms if no potentially traumatic event was reported. The CATS-2 was administered in self-report and caregiver report. PTSS sum scores range from 0 to 60, with sum scores equal to or above 21 indicating clinically relevant PTSS symptoms [71]. Sum scores were utilized for the analyses. Internal consistency in our sample was very good for self-reports (Cronbach’s α = 0.90) and good for caregiver reports (Cronbach’s α = 0.89).

### Rosenberg self-esteem scale (RSES)

The German version of the Rosenberg Self-Esteem Scale (RSES; [74]) was used to assess self-esteem. This Scale uses 10 self-report items with a 6-point Likert scale ranging from 1 = “strongly disagree” to 6 = “strongly agree”. RSES sum scores range from 10 to 60, with sum scores below 41 indicating below average self-esteem [75]. Sum scores were utilized for the analyses. Internal consistency in our sample was good (Cronbach’s α = 0.85).

### Patient health questionnaire 9 (PHQ-9)

The German version of the Patient Health Questionnaire 9 (PHQ-9; [63]) was used to assess depressive symptoms. This Scale uses 9 items with a 4-point Likert scale ranging from 0 = “never” to 3 = “almost every day”. Sum scores range from 0 to 27, with scores equal to or above 11 indicating clinically relevant depressive symptoms in adolescents [76, 77]. Sum scores were utilized for the analyses. Internal consistency in our sample was good (Cronbach’s α = 0.87).

### Feasibility and treatment fidelity

Feasibility was measured via the drop-out rate, session attendance of each participant, and safety (occurrence of serious adverse events). We assessed the following serious adverse events in the context of this study: emergency consultations in child and adolescent psychiatry or with a psychotherapist, absence from the youth welfare institution without permission, hospitalization and suicide attempts or suicide during the period of intervention participation. Serious adverse events had to be reported immediately after their occurrence to the study center by the coordinator of the responsible youth welfare institution. Further actions were then coordinated between the institution and the clinical advisor in the study center. Additionally, several questions and statements regarding the adolescents’ current placement situation and feedback on the intervention were assessed. One of the feedback questions referred to the occurrence of unpleasant situations in the course of the intervention (“Were there any situations in the group sessions that made you feel uncomfortable?“). After answering the initial dichotomous question affirmatively, the corresponding situations could be entered as free text input. Statements referred to the helpfulness of the participation and the recommendation of participating in the intervention to peers. The agreement with these statements was assessed on a 4-point Likert scale ranging from 1 = “does not apply” to 4 = “does fully apply”. Treatment fidelity was assessed using content checklists for each session filled out by the group leaders and sent to the study center on a weekly basis.

### Statistical procedures

To investigate changes in the outcomes and their sustainability, we conducted mixed effect models on the outcome measures using the sample of participants who completed the intervention. First, we only used time (pre-intervention, post-intervention, 3-month follow-up) as fixed effect in the model. In a second step we used time (pre-intervention, post-intervention, 3-month follow-up), duration of stay in the current youth welfare institution, and group (clinically relevant pre-intervention scores versus clinically not relevant pre-intervention scores on YSR, CBCL, PHQ-9 and CATS-2 self-report and caregiver report; average pre-intervention score versus below average pre-intervention score on GSE and RSES) as well as their interactions as fixed effects. We included duration of stay in days in the current institution as a fixed effect due to the wide range of time that participants had lived in the current youth welfare institution in the study sample. This was done to explore for any potential confounding effect of this variable on the changes in the outcomes in the course of the intervention, given its primary development for use at the beginning of an out-of-home placement. Age and gender were integrated into the model for the exploration of moderating effects. We found additional interaction effects between age and time on total problem behavior (*b* = − 0.06, *F*(1, 94) = 4.90, *p* =.029), and between age and time on externalizing behavior problems (*b* = − 0.07, *F*(1, 290) = 3.94, *p* =.048), though only in caregiver reports and with comparably small estimates. All other interactions remained the same regarding their estimates and significance. The factor gender had no moderating effect at all. However, the model fit indices (AIC and BIC) indicated that the model fit was better without the inclusion of age and gender for every outcome. For total problem behavior the inclusion of age and gender in the model increased the AIC value by 8.53 points (609.77 to 618.30) and the BIC value by 8.47 points (624.50 to 632.98). For externalizing behavior problems, the increase of the AIC value was 5.21 points (593.57 to 598.78) and the increase of the BIC value was 5.16 points (608.30 to 613.46). Hence, we decided to not include age and gender into the final model. The study sample was divided into two distinct groups, the high burden group (HB group) and the low burden group (LB group), separately for each outcome. The allocation of every participant to the respective group was determined based on their pre-intervention score and on the specific cut-off of each outcome. The aim here was to investigate whether the intervention is equally suitable for adolescents with a high and low mental health burden.

Mixed effect models were used because they can handle missing data under the missing at random assumption. There were no missing items in the questionnaires because the online tool used for data collection did not allow missing items. However, due to accidentally omitting questionnaires in the online assessment tool, two CBCL questionnaires (less than 2%) and four CATS-2 caregiver reports (less than 4%) in the post-intervention assessment could not be collected. Caregivers, but not adolescents, had the opportunity to enter data at their own convenience using a specific manual, and this procedure failed in a small proportion of cases. Additionally, 3-month follow-up data from seven adolescents who completed the intervention could not be collected because they left the youth welfare institution as planned before the 3-month follow-up assessment. These adolescents either returned to their family of origin in accordance with the helping plan agreed upon with the responsible youth welfare office or reached the age of majority. Parameters were estimated using the restricted maximum likelihood (REML) method. Data were nested by participants because of the longitudinal design of the study, and repeated measures were modeled using an unstructured covariance matrix based on the comparison of likelihood criteria (AIC and BIC). Effect sizes (Cohen’s *d*) were calculated by group for pre to post, pre to 3-month follow-up and post to 3-month follow-up differences using the pooled standard deviation. To estimate the between-group effect sizes, the pooled standard deviation of the difference scores was used. An effect size of|d| = 0.2 indicates a small effect,|d| = 0.5 a medium effect, and|d| = 0.8 a large effect for an intervention [78]. All analyses were carried out using IBM SPSS Version 29.

## Results

### Participant flow and sample description

**Fig. 1:**
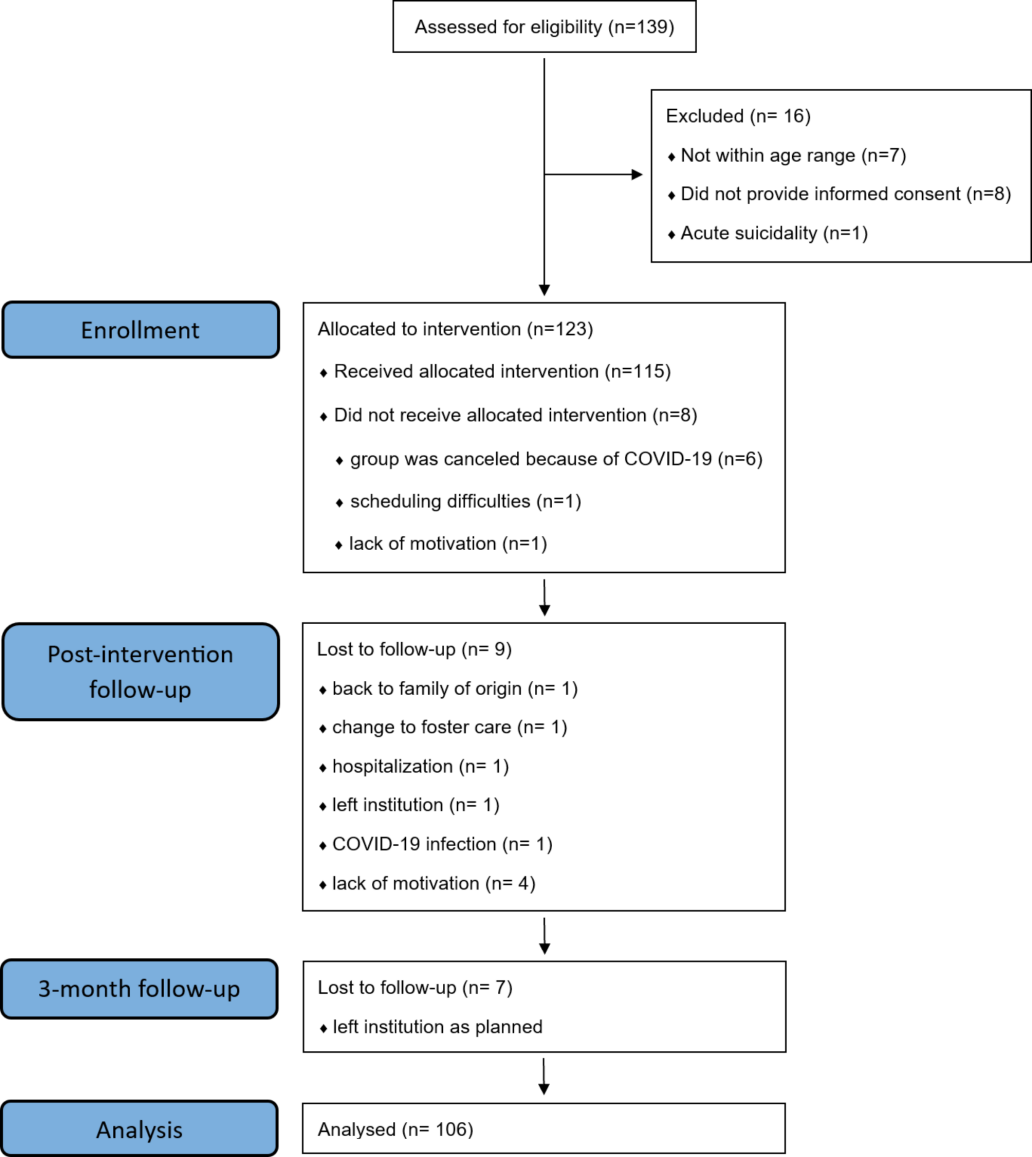
Study flowchart

Figure 1 presents the CONSORT diagram illustrating participant flow. Altogether 139 adolescents were assessed for eligibility. In total, *n* = 16 (11.5%) did not meet the inclusion criteria (for the reasons, see Fig. 1). Therefore, a final sample of *n* = 123 adolescents were enrolled in the study. Differences between participants who were either excluded or included in the study could not be determined due to a lack of informed consent from non-participants. Once allocated, *n* = 115 adolescents started the intervention; *n* = 8 did not (for reasons, see Fig. 1). The mean age of the participants in the intervention was 14.91 years (*SD* = 1.45; ranging from 12.19 to 17.94 years), and *n* = 60 (52.2%) participants were male. The average duration of stay in the current youth welfare institution was 645.15 days (*SD* = 657.21; ranging from 17 to 3577 days). A total of *n* = 9 (7.8%) participants did not complete the intervention (for the reasons, see Fig. 1). Additional details about the hospitalization of one participant are provided in the last paragraph of the “Feasibility and treatment fidelity” section regarding serious adverse events. The reason one participant left the institution before the post-intervention follow-up was due to the termination of his placement resulting from persistent rule-breaking behavior. Hence, the analysed sample consisted of *n* = 106 adolescents who participated in the full intervention. In the sample of participants who did not complete the intervention, there were significantly more female adolescents compared to the sample of participants who completed the intervention (χ²(1) = 6.60, *p* =.010). Additionally, the adolescents who dropped out of the intervention had lived in the current youth welfare institution for a significantly shorter duration than the adolescents who completed the intervention (*t*(26) = −4.09, *p* <.001). There were no significant differences between the sample of participants who completed the intervention and the sample of participants who did not complete the intervention regarding the pre-intervention scores of the questionnaires. Pre-intervention scores of the questionnaires for the participants of the intervention are presented in Table 2. A total of 31 intervention groups were conducted in this study. On average, a group consisted of four participants (range 3–6 participants).

**Table 2:** Pre-intervention scores of the questionnaires, and participants allocated to the high and low burden group

Questionnaire	Total sample (*n* = 115)		High burden group			Low burden group	
	*M* (*SD*); range		*M* (*SD*); range	*n* (%)		*M* (*SD*); range	*n* (%)
GSE	26.47 (6.09); 11.00–40.00		21.46 (3.83); 11.00–25.00	54 (47,0)		30.90 (3.88); 26.00–40.00	61 (53,0)
RSES	41.64 (10.89); 14.00–60.00		30.30 (7.01); 14.00–40.00	44 (38,3)		48.68 (5.63); 41.00–60.00	71 (61,7)
CATS-2 Self	21.13 (11.21); 2.00–50.00		30.04 (7.32); 21.00–50.00	54 (51,4)		11.71 (5.31); 2.00–20.00	51 (48,6)
CATS-2 Care	13.14 (7.75); 0.00–36.00		24.58 (4.48); 21.00–36.00	19 (19,4)		10.39 (5.52); 0.00–20.00	79 (80,6)
YSR Total	63.94 (10.34); 46.00–92.00		69.59 (8.40); 60.00–92.00	73 (63,5)		54.12 (4.21); 46.00–59.00	42 (36,5)
YSR EXT	59.05 (11.07); 37.00–90.00		68.61 (8.68); 60.00–90.00	51 (44,3)		51.44 (5.40); 37.00–59.00	64 (55,7)
YSR INT	61.94 (11.74); 34.00–96.00		69.21 (8.90); 60.00–96.00	67 (58,3)		51.79 (6.54); 34.00–59.00	48 (41,7)
CBCL Total	63.35 (8.83); 44.00–86.00		68.50 (5.99); 60.00–86.00	74 (64,3)		54.05 (4.34); 44.00–59.00	41 (35,7)
CBCL EXT	59.59 (10.50); 36.00–80.00		67.72 (5.40); 60.00–80.00	61 (53,0)		50.41 (6.47); 36.00–59.00	54 (47,0)
CBCL INT	61.77 (10.06); 38.00–86.00		67.94 (6.70); 60.00–86.00	71 (61,7)		51.82 (5.53); 38.00–59.00	44 (38,3)
PHQ-9	7.97 (6.24); 0.00–25.00		15.71 (3.88); 11.00–25.00	35 (30,4)		4.58 (3.39); 0.00–10.00	80 (69,6)

GSE = General Self-Efficacy Scale (sum score); RSES = Rosenberg Self-Esteem Scale (sum score); CATS-2 Self = Child and Adolescent Trauma Screen (self-report; sum score); CATS-2 Care = Child and Adolescent Trauma Screen (caregiver report; sum score); YSR Total = Youth Self Report total score (T-score); YSR INT = Youth Self Report internalizing behavior (T-score); YSR EXT = Youth Self Report externalizing behavior (T-score); CBCL Total = Child Behavior Checklist total score (T-score); CBCL INT = Child Behavior Checklist internalizing behavior (T-score); CBCL EXT = Child Behavior Checklist externalizing behavior (T-score); PHQ-9 = Patient Health Questionnaire (sum score); The allocation of each participant to either the high burden group or the low burden group was determined based on their pre-intervention score and the specific cut-off for each questionnaire.

### Primary outcome

In the first step of analysis, we observed a significant increase in self-efficacy over time (*b* = 0.97, *F*(1, 300) = 12.57, *p* <.001) with a small effect size (*d* = − 0.24) between the pre- and post-intervention assessment. These improvements were stable in the 3-month follow-up period. Detailed estimates, standard errors, and confidence intervals for this model are given in additional file 1 in the online supplement, while effect sizes are presented in additional file 2. The same is true for subsequent sections.

In the second step of the analysis, closer examination of the data revealed a significant interaction between group and time (*b* = 0.37, *F*(1, 296) = 13.07, *p* <.001) indicating an increase in self-efficacy between the pre- and post-intervention assessment in the HB group, but not in the LB group. In both groups, there was no significant improvement or deterioration from post-intervention to 3-month follow-up indicating that the improvements in the HB group were stable in the 3-month follow-up period. The pre-post effect size in the HB group was *d* = − 0.80. The between-group effect size post-intervention was *d* = 1.01. For means, standard deviations, and effect sizes, see Table 3. For estimates, standard errors, and confidence intervals of the extended mixed effect model, see Table 4. The same is true for subsequent sections.

**Table 3:** Means, standard deviations, and effect sizes by group and time (pre-intervention, post-intervention, 3-month follow-up)

	Difference: Pre-post		Difference: Pre-3MFU		Difference: Post-3MFU	
	*M (SD)*	Statistics	*M (SD)*	Statistics	*M (SD)*	Statistics
High burden group						
GSE	− 4.08 (5.09)	***p*** **<.001** ***d*** **= − 0.80**	− 4.13 (5.40)	***p* <.001** ***d*** **= − 0.77**	− 0.04 (5.70)	*p* = 1 *d* = − 0.01
RSES	− 4.19 (6.16)	***p* <.001** ***d*** **= − 0.68**	− 7.51 (9.02)	***p* <.001** ***d* = − 0.83**	− 3.16 (7.93)	*p* =.060 *d* = − 0.40
CATS-2 self	5.33 (9.25)	***p* <.001** ***d*** **= 0.58**	7.50 (10.47)	***p* <.001** ***d*** **= 0.72**	2.44 (9.44)	*p* =.291 *d* = 0.26
CATS-2 care	2.63 (10.72)	*p* = 1*d* = 0.25	5.59 (7.94)	***p* =.031** ***d* = 0.70**	2.94 (6.31)	*p* =.246 *d* = 0.47
YSR total	3.61 (6.51)	***p* <.001** ***d*** **= 0.55**	4.49 (8.42)	***p* <.001** ***d* = 0.53**	1.20 (6.62)	*p* =.489 *d* = 0.18
YSR INT	5.10 (6.91)	***p* <.001** ***d*** **= 0.74**	5.87 (10.31)	***p* <.001** ***d* = 0.57**	1.19 (8.09)	*p* =.861 *d* =0.15
YSR EXT	3.24 (6.22)	***p* =.003** ***d*** **= 0.52**	4.56 (6.86)	***p* <.001** ***d* = 0.66**	0.98 (7.16)	*p* = 1 *d* = 0.14
CBCL total	4.39 (8.17)	***p* <.001** ***d*** **= 0.54**	4.34 (8.56)	***p* <.001** ***d* = 0.51**	0.23 (6.96)	*p* = 1 *d* = 0.03
CBCL INT	5.97 (7.90)	***p* <.001** ***d* = 0.76**	5.20 (8.71)	***p* <.001** ***d* = 0.60**	− 0.28 (7.89)	*p* = 1 *d* = − 0.04
CBCL EXT	3.65 (10.12)	***p* =.036** ***d*** **= 0.36**	3.90 (9.08)	***p* =.012** ***d* = 0.43**	0.04 (6.91)	*p* = 1 *d* = 0.01
PHQ-9	3.77 (4.94)	***p* <.001** ***d*** **= 0.76**	5.73 (5.56)	***p* <.001** ***d* = 1.03**	2.00 (5.36)	*p* =.207 *d* = 0.37
Low burden group						
GSE	0.91 (4.81)	*p* =.461 *d* = 0.19	0.07 (4.51)	*p* = 1 *d* = 0.02	− 0.39 (4.80)	*p* = 1 *d* = − 0.08
RSES	1.23 (7.54)	*p* =.585 *d* = 0.16	*0.35 (7.81)*	*p* = 1 *d* = 0.05	− 1.10 (8.18)	*p* =.885 *d* = − 0.13
CATS-2 self	− 0.69 (8.06)	*p* = 1 *d* = − 0.09	2.22 (5.79)	*p* =.078 *d* = 0.38	2.16 (6.04)	*p* =.108 *d* = 0.36
CATS-2 care	1.12 (4.62)	*p* =.198 *d* = 0.24	1.48 (6.02)	*p* =.228 *d* = 0.25	0.33 (5.98)	*p* = 1 *d* = 0.06
YSR total	0.85 (6.82)	*p* = 1 *d* = 0.12	2.05 (6.93)	*p* =.228 *d* = 0.30	1.37 (6.07)	*p* =.519 *d* = 0.23
YSR INT	− 0.63 (8.69)	*p* = 1 *d* = − 0.07	0.42 (8.75)	*p* = 1 *d* = 0.05	1.20 (7.02)	*p* =.774 *d* = 0.17
YSR EXT	− 0.26 (6.67)	*p* = 1 *d* = − 0.04	0.21 (5.95)	*p* = 1 *d* = 0.04	1.05 (5.43)	*p* =.456 *d* = 0.19
CBCL total	1.39 (4.89)	*p* =.261 *d* = 0.29	1.65 (6.46)	*p* =.387 *d* = 0.26	0.32 (4.44)	*p* = 1 *d* = 0.07
CBCL INT	1.15 (6.76)	*p* =.879 *d* = 0.17	− 0.34 (6.39)	*p* = 1 *d* = − 0.05	− 1.11 (4.99)	*p* =.555 *d* = − 0.22
CBCL EXT	0.65 (6.38)	*p* = 1 *d* = 0.10	0.32 (8.29)	*p* = 1 *d* = 0.04	0.08 (7.58)	*p* = 1 *d* = 0.01
PHQ-9	− 0.62 (4.62)	*p* =.741 *d* = − 0.13	− 0.12 (4.16)	*p* = 1 *d* = − 0.03	0.81 (4.93)	*p* =.495 *d* = 0.16
Between-group effect size						
GSE	*d* = 1.01		*d* = 0.85		*d* = − 0.07	
RSES	*d* = 0.77		*d* = 0.95		*d* = 0.26	
CATS-2 Self	*d* = − 0.69		*d* = − 0.61		*d* = − 0.04	
CATS-2 care	*d* = − 0.24		*d* = − 0.63		*d* = − 0.43	
YSR total	*d* = − 0.42		*d* = − 0.31		*d* = 0.03	
YSR INT	*d* = − 0.74		*d* = − 0.57		*d* = 0.002	
YSR EXT	*d* = − 0.54		*d* = − 0.68		*d* = 0.01	
CBCL total	*d* = − 0.42		*d* = − 0.34		*d* =0.02	
CBCL INT	*d* = − 0.64		*d* = − 0.70		*d* = − 0.12	
CBCL EXT	*d* = − 0.35		*d* = − 0.41		*d* = 0.01	
PHQ-9	*d* = − 0.93		*d* = − 1.28		*d* = − 0.24	

3MFU = 3-month follow-up; GSE = General Self-Efficacy Scale (sum score); RSES = Rosenberg Self-Esteem Scale (sum score); CATS-2 Self = Child and Adolescent Trauma Screen (self-report; sum score); CATS-2 Care = Child and Adolescent Trauma Screen (caregiver report; sum score); YSR Total = Youth Self Report total score (T-score); YSR INT = Youth Self Report internalizing behavior (T-score); YSR EXT = Youth Self Report externalizing behavior (T-score); CBCL Total = Child Behavior Checklist total score (T-score); CBCL INT = Child Behavior Checklist internalizing behavior (T-score); CBCL EXT = Child Behavior Checklist externalizing behavior (T-score); PHQ-9 = Patient Health Questionnaire (sum score); all p-values Bonferroni corrected; The allocation of each participant to either the high burden group or the low burden group was determined based on their pre-intervention score and the specific cut-off for each questionnaire.

**Table 4:** Results of the mixed effects models with time, group, and duration as fixed effects

Outcome	Estimates of fixed effects			
	Estimate (*b*)	*SE* (*b*)	95% *CI*	*p*
GSE				
Intercept	0.47	0.09	0.29, 0.64	**<.001**
Time	− 0.02	0.07	− 0.15, 0.12	.791
Group	− 1.34	0.14	− 1.61, − 1.07	**<.001**
Time x Group	0.37	0.10	0.17, 0.57	**<.001**
Duration	0.08	0.07	− 0.06, 0.21	.253
Duration x Time	0.01	0.05	− 0.09, 0.11	.791
RSES				
Intercept	0.50	0.08	0.34, 0.65	**<.001**
Time	0.004	0.05	− 0.09, 0.10	.931
Group	− 1.63	0.12	− 1.87, − 1.38	**<.001**
Time x group	0.36	0.08	0.20, 0.52	**<.001**
Duration	0.10	0.06	− 0.02, 0.22	.086
Duration x Time	0.03	0.04	− 0.04, 0.11	.374
CATS-2 Self				
Intercept	− 0.57	0.08	− 0.73, − 0.41	**<.001**
Time	− 0.07	0.07	− 0.20, 0.06	.282
Group	1.50	0.12	1.27, 1.73	**<.001**
Time x group	− 0.28	0.09	− 0.47, − 0.09	**.003**
Duration	− 0.15	0.06	− 0.27, − 0.03	**.013**
Duration x time	0.01	0.05	− 0.08, 0.11	.819
CATS-2 care				
Intercept	− 0.23	0.08	− 0.38, − 0.08	**.004**
Time	− 0.08	0.05	− 0.18, 0.02	.133
Group	1.76	0.18	1.40, 2.12	**<.001**
Time x group	− 0.25	0.11	− 0.47, − 0.03	**.024**
Duration	− 0.07	0.07	− 0.21, 0.06	.285
Duration x time	− 0.01	0.05	− 0.10, 0.08	.761
YSR total				
Intercept	− 0.70	0.11	− 0.92, − 0.47	**<.001**
Time	− 0.09	0.06	− 0.21, 0.03	.123
Group	1.39	0.15	1.10, 1.68	**<.001**
Time x group	− 0.13	0.08	− 0.28, 0.03	.107
Duration	0.04	0.07	− 0.10, 0.18	.578
Duration x time	− 0.01	0.04	− 0.09, 0.06	.722
YSR externalizing				
Intercept	− 0.55	0.09	− 0.72, − 0.37	**<.001**
Time	− 0.01	0.04	− 0.09, 0.07	.724
Group	1.49	0.14	1.22, 1.76	**<.001**
Time x group	− 0.20	0.06	− 0.32, − 0.08	**.001**
Duration	− 0.01	0.07	− 0.15, 0.12	.849
Duration x time	− 0.02	0.03	− 0.08, 0.04	.441
YSR Internalizing				
Intercept	− 0.62	0.11	− 0.83, − 0.41	**<.001**
Time	− 0.03	0.06	− 0.15, 0.09	.659
Group	1.43	0.14	1.14, 1.71	**<.001**
Time x group	− 0.25	0.08	− 0.41, − 0.09	**.003**
Duration	− 0.11	0.07	− 0.25, 0.03	.123
Duration x time	0.03	0.04	− 0.05, 0.11	.424
CBCL total				
Intercept	− 0.75	0.09	− 0.94, − 0.56	**<.001**
Time	− 0.08	0.07	− 0.21, 0.06	.282
Group	1.46	0.12	1.22, 1.69	**<.001**
Time x group	− 0.16	0.09	− 0.34, 0.01	.066
Duration	0.03	0.06	− 0.09, 0.14	.651
Duration x time	− 0.01	0.04	− 0.09, 0.07	.826
CBCL externalizing				
Intercept	− 0.64	0.07	− 0.79, − 0.50	**<.001**
Time	− 0.02	0.07	− 0.15, 0.12	.785
Group	1.47	0.10	1.27, 1.68	**<.001**
Time x group	− 0.15	0.10	− 0.34, 0.04	.125
Duration	− 0.01	0.05	− 0.09, 0.11	.862
Duration x time	− 0.04	0.05	− 0.13, 0.06	.420
CBCL Internalizing				
Intercept	− 0.79	0.11	− 1.00, − 0.58	**<.001**
Time	0.02	0.07	− 0.11, 0.16	.744
Group	1.52	0.13	1.26, 1.78	**<.001**
Time x group	− 0.31	0.09	− 0.49, − 0.14	**<.001**
Duration	− 0.03	0.06	− 0.16, 0.09	.591
Duration x Time	− 0.02	0.04	− 0.10, 0.06	.662
PHQ-9				
Intercept	1.46	0.11	1.24, 1.68	**<.001**
Time	− 0.50	0.08	− 0.67, − 0.34	**<.001**
Group	1.84	0.13	1.58, 2.10	**<.001**
Time x group	− 0.48	0.10	− 0.68, − 0.29	**<.001**
Duration	− 0.06	0.06	− 0.17, 0.06	.357
Duration x time	− 0.03	0.04	− 0.11, 0.06	.515

GSE = General Self-Efficacy Scale; RSES = Rosenberg Self-Esteem Scale; CATS-2 Self = Child and Adolescent Trauma Screen (self-report); CATS-2 Care = Child and Adolescent Trauma Screen (caregiver report); YSR Total = Youth Self Report total score; YSR INT = Youth Self Report internalizing behavior; YSR EXT = Youth Self Report externalizing behavior; CBCL Total = Child Behavior Checklist total score; CBCL INT = Child Behavior Checklist internalizing behavior; CBCL EXT = Child Behavior Checklist externalizing behavior; PHQ-9 = Patient Health Questionnaire.

### Secondary outcomes

#### Rosenberg self-esteem scale (RSES)

In the first step of analysis, we observed a significant increase in self-esteem over time (*b* = 1.58, *F*(1, 101) = 12.83, *p* =.001) with a small effect size (*d* = − 0.28) between the pre-intervention and the 3-month follow-up assessment, but not between the pre- and post-intervention assessment.

Closer examination of the data in the second step of the analysis revealed a significant interaction between group and time (*b* = 0.36, *F*(1, 98) = 20.96, *p* <.001) indicating an increase in self-esteem between the pre- and post-intervention assessment in the HB group, but not in the LB group. In the LB group, there was no statistically significant improvement or deterioration at any time. The pre-post effect size in the HB group was *d* = − 0.68, and the improvement was stable in the 3-month follow-up period. The between-group effect size post-intervention was *d* = 0.77.

### Child and adolescent trauma screen version 2 (CATS-2)

In total, *n* = 90 adolescents (84.9%) self-reported at least one potentially traumatic life event. The caregivers reported known potentially traumatic life events for *n* = 76 adolescents (71.7%). In the first step of analysis, we observed a significant decrease in self-reported PTSS over time (*b* = − 2.41, *F*(1, 85) = 24.44, *p* <.001) with a small effect size between the pre- and post-intervention assessment (*d* = 0.29), as well as between the post-intervention and 3-month follow-up assessment (*d* = 0.28). In caregiver reports we observed a significant decrease in PTSS over time (*b* = − 1.01, *F*(1, 78) = 6.58, *p* =.012) with a small effect size (*d* = 0.37) between the pre-intervention and 3-month follow-up assessment.

Closer examination of the data in the second step of the analysis revealed a significant interaction between group and time regarding self-reported PTSS (*b* = − 0.28, *F*(1, 260) = 8.77, *p* =.003) and PTSS in caregiver reports (*b* = − 0.25, *F*(1, 69) = 5.32, *p* =.024) indicating a decrease in PTSS over time in the HB group, but not in the LB group. The pre-post effect size for the decrease in self-reported PTSS in the HB group was *d* = 0.58. There was no significant improvement or deterioration in self-reported PTSS between the post-intervention and 3-month follow-up assessment indicating that the improvements were stable in the 3-month follow-up period. In caregiver reports, only the decrease in PTSS between the pre-intervention and 3-month follow-up assessment in the HB group was found to be significant (*d* = 0.70). The between-group effect size in self-reported PTSS post-intervention was *d* = − 0.69. The between-group effect size in caregiver-reported PTSS for the pre-intervention and 3-month follow-up difference was *d* = − 0.63. The analyses also revealed a significant inverse relationship between time spent in the current youth welfare institution and self-reported PTSS, indicating that adolescents who had spent more time in residential care exhibited fewer PTSS pre-intervention (*b* = − 0.15, *F*(1, 260) = 6.20, *p* =.013).

### Patient health questionnaire 9 (PHQ 9)

In the first step, we observed a significant decrease in depressive symptoms over time (*b* = − 0.92, *F*(1, 300) = 12.80, *p* <.001) with a small effect size (*d* = 0.27) between the pre-intervention and 3-month follow-up assessment.

In the second step of the analysis, closer examination of the data revealed a significant interaction between group and time (*b* = − 0.48, *F*(1, 296) = 23.55, *p* <.001) indicating a decrease in depressive symptoms between the pre- and post-intervention assessment in the HB group, but not in the LB group. The pre-post effect size in the HB group was *d* = 0.76. In both groups, there was no significant decrease or increase in depressive symptoms between the post-intervention and 3-month follow-up assessment indicating that the decrease in depressive symptoms in the HB group was stable in the 3-month follow-up period. The between-group effect size post-intervention was *d* = − 0.93.

### Achenbach scales

In the first step of analysis, we observed a significant decrease in self-reported internalizing behavior problems over time (*b* = − 1.96, *F*(1, 98) = 15.27, *p* <.001) with a small effect size between the pre- and post-intervention assessment (*d* = 0.32). This decrease was found to be stable during the 3-month follow-up period. For caregiver reports we observed a similar pattern with a significant decrease in internalizing behavior problems over time (*b* = − 1.71, *F*(1, 98) = 16.16, *p* <.001) with a medium effect size (*d* = 0.53) between the pre- and post-intervention assessment. This decrease was also found to be stable during the 3-month follow-up period.

Regarding externalizing behavior problems, we observed a significant decrease in self-reported externalizing problem behavior over time (*b* = − 1.10, *F*(1, 99) = 10.47, *p* =.002) with a small effect size (*d* = 0.31) between the pre-intervention and 3-month follow-up assessment. For caregiver reports we observed a significant decrease in externalizing problem behavior over time (*b* = − 1.06, *F*(1, 99) = 5.54, *p* =.021) with a small effect size (*d* = 0.25) between the pre- and post-intervention assessment. This decrease was found to be stable during the 3-month follow-up period.

When looking at self-reported total problem behavior, we observed a significant decrease over time (*b* = − 1.90, *F*(1, 97) = 22.02, *p* <.001) with a small effect size (*d* = 0.39) between the pre- and post-intervention assessment. This decrease was found to be stable during the 3-month follow-up period. For caregiver reports we observed a similar pattern with a significant decrease in total problem behavior over time (*b* = − 1.74, *F*(1, 98) = 18.30, *p* <.001) with a small effect size (*d* = 0.45) between the pre- and post-intervention assessment. This decrease was also found to be stable during the 3-month follow-up period.

In the second step of the analysis, closer examination of the data revealed a significant interaction between group and time in self-reports (*b* = − 0.25, *F*(1, 96) = 9.39, *p* =.003) and caregiver reports (*b* = − 0.31, *F*(1, 294) = 12.45, *p* <.001) of internalizing behavior problems indicating a decrease in internalizing behavior problems between the pre- and post-intervention assessment in the HB group, but not in the LB group. The self-reported pre-post effect size in the HB group was *d* = 0.74. For caregiver reports the pre-post effect size in the HB group was *d* = 0.76. In the LB group, there was no significant improvement or deterioration at any time. The between-group effect size post-intervention was *d* = − 0.74 for self-report and *d* = − 0.64 for caregiver report.

When looking at externalizing behavior problems, we observed a similar pattern in self-reports, with a significant interaction between group and time (*b* = − 0.20, *F*(1, 296) = 10.36, *p* =.001) indicating a decrease in externalizing behavior problems between the pre- and post-intervention assessment in the HB group, but not in the LB group. The self-reported pre-post effect size in the HB group was *d* = 0.52. The between-group effect size post-intervention for self-report was *d* = − 0.54. In caregiver reports no significant interaction between group and time was observed (*b* = − 0.15, *F*(1, 294) = 2.37, *p* =.125). No significant interaction of group and time was observed for the total problem behavior score either in self-reports (*b* = − 0.13 *F*(1, 94) = 2.65, *p* =.107) or in caregiver reports (*b* = − 0.16, *F*(1, 94) = 3.45, *p* =.066).

### Feasibility and treatment fidelity

The rate of enrolled adolescents who actually started the intervention was 93.5%, and the most frequent reason for not participating at all was canceling of the group due to COVID-19 (see Fig. 1). Furthermore, 92.2% of the participants who received the intervention completed participation (see Fig. 1). Attendance in the group sessions was also high, with 75.5% (*n* = 80) of the participants attending all intervention sessions, 14.2% (*n* = 15) missing one session, 7.6% (*n* = 8) missing two sessions, and 2.8% (*n* = 3) missing more than two sessions. If participants missed individual sessions, the group leaders planned an extra session with them, to cover the missed content, before the next scheduled session.

In the dichotomous feedback questions asked anonymously at the post-intervention assessment, *n* = 81 (76.4%) participants stated that they did not recall feeling uncomfortable during group sessions. The most frequently mentioned reason for feeling uncomfortable during group sessions was associated with talking about the past (*n* = 8; 7.6%). The second most frequently mentioned reason for feeling uncomfortable during group sessions was also related to the past. More specifically, it was associated with the perceived stress regarding the biographical topics of some sessions in general (*n* = 7; 6.6%). There was a wide range of other individual mentions by participants (*n* = 10; 9.4%) such as tiredness, discomfort during role-plays or lack of time for participation.

The high acceptance of the intervention program was also indicated by the ratings of helpfulness of the participation and the recommendation of participation to peers: 75.5% of the participants (*M* = 2.97, *SD* = 0.88) rated participation as helpful in general (scores ≥ 3), and 89.4% (*M* = 3.31, *SD* = 0.87) recommended participation in the intervention to their peers (scores ≥ 3). Furthermore, 84.3% (*M* = 2.97, *SD* = 0.57) of the caregivers who provided the caregiver reports rated the participation as beneficial for the adolescents (scores ≥ 3).

Regarding treatment fidelity, 96.2% of the content of the intervention was marked as completed within the session protocols. Regarding safety, only three incidents occurred that had to be classified as serious adverse events: One participant was referred to inpatient treatment because of a longer-lasting substance abuse problem and, therefore, had to abandon participation. Two other participants had an emergency consultation in a psychiatric clinic due to a suicidal crisis, which had no traceable relation to their participation in the intervention. In both cases, inpatient treatment was not required, and the participants completed the intervention.

## Discussion

This is the first study to investigate the feasibility and potential effectiveness of a manualized group intervention based on life story work for adolescents in German youth welfare institutions. Findings from this study indicate that participation in the ANKOMMEN intervention could contribute to enhancing self-efficacy and self-esteem in those participants who had below average pre-intervention scores. Moreover, the intervention could contribute to the reduction of self-reported and caregiver-reported internalizing behavior problems, self-reported externalizing behavior problems, self-reported PTSS, and self-reported depressive symptoms, for those participants with clinically relevant symptoms prior to the intervention. All of these improvements were stable in the 3-month follow-up period.

As outlined in the introduction to this paper, there are various mechanisms in the context of life story work and identity development, as well as in the context of group interventions in general, that can serve as explanations for the measured enhancement of self-efficacy and self-esteem. In addition, the enhancement of self-esteem might be also a result of writing and talking about important life story chapters. Steiner, Pillemer and Thomsen [79] showed in three experimental studies that writing about important life chapters increases self-esteem ratings. They suggested that writing and reflecting on negative experiences could lead to the habituation of associated negative emotions, potentially resulting in feelings of mastery that increase self-esteem [79]. Habituation is a known mechanism in trauma-focused treatments, where patients are exposed to their traumatic experiences using narrative techniques. In ANKOMMEN, participants wrote narratives about the critical life events of leaving their homes and starting a new life in residential care which means that the reduction in PTSS could also be explained by this mechanism. The decrease in the depressive symptoms of participants with clinically relevant symptoms prior to the intervention in this study is a good fit with the concept of life story work and its main goal of helping to develop a cohesive identity. Hallford, Ricarte and Hermans [80] showed that higher causal coherence, which can be defined as the general understanding of how life experiences are associated with one another, predicts higher self-efficacy and self-esteem. In the study by Hallford, Ricarte and Hermans [80], the latent construct ‘positive self-concept,’ which is a combination of self-efficacy and self-esteem, predicted a significant decrease in depressive symptoms. The relationships outlined before could explain the reduction in depressive symptoms observed in this study. The effect sizes of the decrease in depressive symptoms in this study were similar to those of a trauma-focused group intervention, which is also conducted by social workers in residential care [81]. Besides these specific explanations, it is possible that the guided reflection on critical life experiences in the intervention group and the learning of strategies for emotion regulation and problem-solving facilitated the use of active coping strategies (e.g., confrontation with adverse emotions and experiences or seeking social support) in participants in general, which is also associated with increases in self-esteem and decreases in depressive symptoms [82].

The improvements in internalizing and externalizing behavior problems observed in this study are important because some research findings indicate that most of the children and adolescents in residential care did not show any significant decrease in internalizing and externalizing problem behavior over time [83, 84]. However, a study in Switzerland [5] showed similar decreases in internalizing and externalizing behavior problems for children and adolescents in residential care with clinically relevant problems over a time period of more than 9 months. The children and adolescents in that study received standard care, but no targeted intervention such as ANKOMMEN. This indicates that ANKOMMEN might support the recovery process in residential care and could therefore boost placement stability and a positive long-term prognosis, because the improvements in the course of the intervention were stable three months after participation.

The findings of the study also indicate that adolescents with clinically irrelevant behavioral problems, depressive symptoms, or PTSS, as well as those with at least average self-efficacy and self-esteem scores prior to the intervention, did not show significant changes through participation in the corresponding measures. One possible explanation for this finding is that the use of functional coping strategies even prior to the intervention caused only minimal psychological strain in daily life and therefore little need and margin for improvements. These adolescents might have benefited from participation in other elements of life story work, such as better self-understanding, identity development or improvement of relationships (see [30]), which were not explicitly assessed with quantitative measures in this study. However, we found indications of such processes in the qualitative analyses of the interviews with participants of ANKOMMEN [61]. In combination with the results regarding the safety of the intervention, the conclusion can be drawn that participation in the ANKOMMEN intervention might be beneficial for adolescents with elevated mental health burdens but is also suitable for adolescents with low mental health burdens. The analysis also revealed that the time spent in the current youth welfare institution had no significant effect on the measured improvements in the course of the intervention. This indicates that adolescents may benefit from the intervention independent of the duration of their placement in the current child welfare institution and therefore no restriction of application to a certain time period is needed.

The intervention also proved its feasibility in standard care. The high completion rates of the intervention, the high attendance in the group sessions, and the positive evaluative statements of the adolescents are indicators of sufficient feasibility and acceptance. The high rate of treatment fidelity and the safety of the intervention indicates that the intervention can be applied successfully by social workers who were supplied with a manual, training, and case consultations. This is of particular importance as the implementation of the intervention by social workers could help to buffer the lack of targeted mental health support by mental health specialists in the area of youth welfare and improve the accessibility of the benefits of life story work for burdened adolescents in care. These findings are of major importance because during the development of the intervention, there was sometimes uncertainty within the cooperating institutions about whether addressing burdening life experiences and memories in group sessions without further treatment could even increase behavioral problems and PTSS. However, the results of this study clearly indicate that the ANKOMMEN intervention provided a safe framework to address stressful life experiences and could strengthen burdened participants without causing any harm or increasing their mental health burden.

### Limitations

There are some limitations of this study. The most important limitation is the lack of a randomized control condition. Thus, the changes measured in the group of adolescents with a higher mental health burden cannot be attributed to the intervention with sufficient confidence. A subsequent randomized controlled trial is essential to draw more valid conclusions regarding the intervention’s effects and prevent false interpretation of effects of confounding variables, such as standard care in youth welfare programs, effects of other interventions, maturation effects, and regression to the mean in the questionnaires, as effects of participation in the intervention. In our study sample, 42.2% of the participants had at least one additional session with a psychotherapist between the pre-intervention screening and the post-intervention follow-up, and 40.4% between the post-intervention follow-up and the 3-month follow-up. This is not unusual, as the opportunity for psychotherapeutic treatment is part of the standard care in some youth welfare institutions. However, it could have confounded the treatment effects of our intervention, particularly in the high-burden group.

Moreover, it is possible that the intervention training for social workers generally raised awareness of topics and methods of life story work or strategies for dealing with adverse emotions and stigmatization, and this resulted in more support in these areas outside the intervention setting, too. Especially for adolescents who showed more behavioral problems and had more deficits in the areas of self-efficacy and self-esteem prior to the intervention, the disclosure of personal information in the group may have led to increased positive attention, further conversations, and additional support outside the intervention setting. This could, therefore, have contributed to strengthening experiences or deepening positive relationships with social workers or peers in the institution. Another limitation is related to the time period of the data collection: all data were collected during the COVID-19 pandemic. There are some indications that in up to 40% percent of the children and adolescents in residential care, well-being decreased during the pandemic and that almost one-third of them reported more depressive symptoms [85]. A potential bias due to the pandemic cannot be ruled out and may require further investigation. Moreover, assessing other important aspects of life story work, such as resilience, identity development, or relationship development, could provide valuable insights about additional effects of the intervention and therefore should be part of further investigations. Finally, the follow-up period of three months was relatively short. Future research should envisage longer follow-up periods, potentially extending into adulthood, to generate a more comprehensive understanding of the potential long-lasting effects of this life story approach.

## Conclusion

The findings presented in this study provide preliminary evidence for the feasibility and potential effectiveness of the ANKOMMEN group intervention. The results showed that adolescents in residential care with low self-efficacy and self-esteem, as well as those with clinically relevant internalizing and externalizing behavior problems, depressive symptoms and PTSS, improved in corresponding outcomes after participation in the intervention. These findings highlight the potential of manualized group interventions focusing on processing and coping with experiences associated with an out-of-home placement for adolescents living in residential care. The feasibility of the intervention with social workers in youth welfare institutions widens the range of application of life story work in residential care and could thereby contribute to making its positive effects accessible to more young people. Moreover, it could serve as a starting point for adolescents in care for further exploration of their own biography and identity development, as well as providing an opportunity to enhance the knowledge of social workers in youth welfare institutions working with the life stories of adolescents. Further research is needed to obtain valid evidence for the efficacy of the intervention presented here and for a more detailed investigation of the potential of manualized group interventions based on life story work for adolescents living in residential care in general.

## Electronic supplementary material

Below is the link to the electronic supplementary material.

Supplementary Material 1. Table with the results of the Mixed Effects Models with Time as Fixed Effect.

Supplementary Material 2. Table with Means, Standard Deviations, and Effect Sizes by Time (pre-intervention, post-intervention, 3-month follow-up).

## Data Availability

The datasets used and/or analysed during the current study are available from the corresponding author on reasonable request.
