# Supplementary material for: Evaluation of ANKOMMEN as a group intervention based on life story work for adolescents in residential care in Germany: a single-arm pilot study
Source: Child Adolesc Psychiatry Ment Health. 2024 Oct 22;18:135. doi: 10.1186/s13034-024-00817-w (PMC11515701; doi:10.1186/s13034-024-00817-w)
Supplement: Supplementary file 1 — Supplementary Material 1. Table with the results of the Mixed Effects Models with Time as Fixed Effect. [file 13034_2024_817_MOESM1_ESM.docx]

| **Additional file 1** Results of the Mixed Effects Models with Time as Fixed Effect | | | | |
| --- | --- | --- | --- | --- |
|  | Estimates of Fixed Effects | | | |
| Outcome | Estimate (b) | *SE* (b) | 95% *CI* | *p* |
| GSE |  |  |  |  |
| Intercept | 26.36 | .58 | 25.23, 27.50 | **< .001** |
| Time | .97 | .27 | .43, 1.51 | **< .001** |
| RSES |  |  |  |  |
| Intercept | 41.01 | 1.09 | 38.85, 43.16 | **< .001** |
| Time | 1.58 | .44 | .71, 2.46 | **.001** |
| CATS-2 Self |  |  |  |  |
| Intercept | 20.44 | 1.10 | 18.26, 22.61 | **< .001** |
| Time | -2.41 | .49 | -3.38, -1.44 | **< .001** |
| CATS-2 Care |  |  |  |  |
| Intercept | 12.63 | .83 | 10.97, 14.28 | **< .001** |
| Time | -1.01 | .39 | -1.80, -.23 | **.012** |
| YSR total |  |  |  |  |
| Intercept | 63.41 | 1.06 | 61.32, 65.51 | **< .001** |
| Time | -1.90 | .40 | -2.70, -1.09 | **< .001** |
| YSR Externalising |  |  |  |  |
| Intercept | 58.46 | 1.09 | 56.30, 60.63 | **< .001** |
| Time | -1.10 | .34 | -1.78, -.43 | **.002** |
| YSR Internalising |  |  |  |  |
| Intercept | 61.60 | 1.20 | 59.23, 63.97 | **< .001** |
| Time | -1.96 | .50 | -2.95, -.96 | **< .001** |
| CBCL total |  |  |  |  |
| Intercept | 62.59 | .85 | 60.91, 64.28 | **< .001** |
| Time | -1.74 | .41 | -2.55, -.93 | **< .001** |
| CBCL Externalising |  |  |  |  |
| Intercept | 58.63 | 1.02 | 56.62, 60.65 | **< .001** |
| Time | -1.06 | .45 | -1.95, -.17 | **.021** |
| CBCL Internalising |  |  |  |  |
| Intercept | 61.09 | .95 | 59.20, 62.98 | **< .001** |
| Time | -1.71 | .43 | -2.56, -.87 | **< .001** |
| PHQ-9 |  |  |  |  |
| Intercept | 7.97 | .61 | 6.77, 9.17 | **< .001** |
| Time | -.92 | .26 | -1.42, -.41 | **< .001** |
| *Note.* GSE = General Self-Efficacy Scale; RSES = Rosenberg Self-Esteem Scale; CATS-2 Self = Child and Adolescent Trauma Screen (self-report); CATS-2 Care = Child and Adolescent Trauma Screen (caregiver report); YSR Total = Youth Self Report total score; YSR INT = Youth Self Report internalizing behavior; YSR EXT = Youth Self Report externalizing behavior; CBCL Total = Child Behavior Checklist total score; CBCL INT = Child Behavior Checklist internalizing behavior; CBCL EXT = Child Behavior Checklist externalizing behavior; PHQ-9 = Patient Health Questionnaire. | | | | |
