# Supplementary material for: Evaluation of ANKOMMEN as a group intervention based on life story work for adolescents in residential care in Germany: a single-arm pilot study
Source: Child Adolesc Psychiatry Ment Health. 2024 Oct 22;18:135. doi: 10.1186/s13034-024-00817-w (PMC11515701; doi:10.1186/s13034-024-00817-w)
Supplement: Supplementary file 2 — Supplementary Material 2. Table with Means, Standard Deviations, and Effect Sizes by Time (pre-intervention, post-intervention, 3-month follow-up). [file 13034_2024_817_MOESM2_ESM.docx]

| **Additional file 2** Means, Standard Deviations, and Effect Sizes by Time (pre-intervention, post-intervention, 3-month follow-up) | | | | | | | | |
| --- | --- | --- | --- | --- | --- | --- | --- | --- |
|  | Difference: Pre-Post | |  | Difference: Pre-3MFU | |  | Difference: Post-3MFU | |
|  | *M (SD)* | Statistics |  | *M (SD)* | Statistics |  | *M (SD)* | Statistics |
|  |  |  |  |  |  |  |  |  |
| GSE | -1.35 (5.52) | **p = .040 d = -.24** |  | -1.84 (5.34) | **p = .003 d = -.34** |  | -0.23 (5.20) | p = 1 d = -.04 |
| RSES | -0.92 (7.48) | p = .633 d = -.12 |  | -2.59 (9.08) | **p = .017 d = -.28** |  | -1.87 (8.11) | p = .072 d = -.23 |
| CATS-2 Self | 2.60 (8.99) | **p = .019 d = .29** |  | 5.04 (8.84) | **p < .001 d = .57** |  | 2.24 (7.87) | **p = .034 d = .28** |
| CATS-2 Care | 1.43 (6.34) | p = .156 d = .23 |  | 2.46 (6.70) | **p = .008 d = .37** |  | 0.68 (6.51) | p = 1 d = .10 |
| YSR Total | 2.59 (6.73) | **p < .001 d = .39** |  | 3.56 (7.93) | **p < .001 d = .45** |  | 1.26 (6.38) | p = .156 d = .20 |
| YSR INT | 2.61 (8.21) | **p = .004 d = .32** |  | 3.39 (9.96) | **p = .003 d = .34** |  | 1.19 (7.59) | p = .364 d = .16 |
| YSR EXT | 1.23 (6.68) | p = .185 d = .18 |  | 2.10 (6.69) | **p = .007 d = .31** |  | 1.02 (6.20) | p = .315 d = .16 |
| CBCL Total | 3.30 (7.26) | **p < .001 d = .45** |  | 3.33 (7.91) | **p < .001 d = .42** |  | 0.27 (6.10) | p = 1 d = .04 |
| CBCL INT | 4.16 (7.82) | **p < .001 d = .53** |  | 3.07 (8.31) | **p = .001 d = .37** |  | -0.60 (6.91) | p = 1 d = -.09 |
| CBCL EXT | 2.15 (8.56) | **p = .035 d = .25** |  | 2.09 (8.83) | p = .061 d = .24 |  | 0.06 (7.22) | p = 1 d = .01 |
| PHQ-9 | 0.62 (5.09) | p = .632 d = .12 |  | 1.41 (5.22) | **p = .025 d = .27** |  | 1.12 (5.04) | p = .088 d = .22 |
| *Note.* 3MFU = 3-month follow-up; GSE = General Self-Efficacy Scale (sum score); RSES = Rosenberg Self-Esteem Scale (sum score); CATS-2 Self = Child and Adolescent Trauma Screen (self-report; sum score); CATS-2 Care = Child and Adolescent Trauma Screen (caregiver report; sum score); YSR Total = Youth Self Report total score (T-score); YSR INT = Youth Self Report internalizing behavior (T-score); YSR EXT = Youth Self Report externalizing behavior (T-score); CBCL Total = Child Behavior Checklist total score (T-score); CBCL INT = Child Behavior Checklist internalizing behavior (T-score); CBCL EXT = Child Behavior Checklist externalizing behavior (T-score); PHQ-9 = Patient Health Questionnaire (sum score); all p-values Bonferroni corrected. | | | | | | | | |
